# Similar Clinical Course and Significance of Circulating Innate and Adaptive Immune Cell Counts in STEMI and COVID-19

**DOI:** 10.3390/jcm9113484

**Published:** 2020-10-28

**Authors:** Elena de Dios, Cesar Rios-Navarro, Nerea Perez-Sole, Jose Gavara, Victor Marcos-Garces, Enrique Rodríguez, Arturo Carratalá, Maria J. Forner, Jorge Navarro, Maria L. Blasco, Elvira Bondia, Jaime Signes-Costa, Jose M. Vila, Maria J. Forteza, Francisco J. Chorro, Vicente Bodi

**Affiliations:** 1 Centro de Investigación Biomédica en Red-Cardiovascular (CIBER-CV), 28029 Madrid, Spain; elenaddll@gmail.com (E.d.D.); francisco.j.chorro@uv.es (F.J.C.); 2 Medicine Department, Faculty of Medicine, University of Valencia, 46010 Valencia, Spain; Maria.Jose.Forner@uv.es; 3 Institute of Health Research-INCLIVA, 46010 Valencia, Spain; cesar_rios1@hotmail.com (C.R.-N.); neere_8@hotmail.com (N.P.-S.); jose_4_6_90@hotmail.com (J.G.); rodriguez_enr@gva.es (E.R.); carratala_art@gva.es (A.C.); jorgenavper@gmail.com (J.N.); blasco_luicor@gva.es (M.L.B.); jaimesignescosta@gmail.com (J.S.-C.); jose.m.salinas@uv.es (J.M.V.); 4 Cardiology Department, Hospital Clínico Universitario, 46010 Valencia, Spain; vic_mg_cs@hotmail.com; 5 Biochemical Department, Hospital Clínico Universitario, 46010 Valencia, Spain; 6 Internal Medicine Department, Hospital Clínico Universitario, 46010 Valencia, Spain; 7 Medical Directory, Hospital Clínico Universitario, 46010 Valencia, Spain; 8 Medical Intensive Care Unit, Hospital Clínico Universitario, 46010 Valencia, Spain; 9 Pneumology Service, Hospital Clínico Universitario, 46010 Valencia, Spain; elvirabonre@gmail.com; 10 Physiology Department, Faculty of Medicine, University of Valencia, 46010 Valencia, Spain; 11 Cardiovascular Medicine Unit, Center of Molecular Medicine, Department of Medicine, Karolinska Institutet, Karolinska University Hospital, 171 77 Stockholm, Sweden; maria.forteza.de.los.reyes@ki.se

**Keywords:** COVID-19, myocardial infarction, neutrophils, lymphocyte, prognosis, severity

## Abstract

This study aimed to assess the time course of circulating neutrophil and lymphocyte counts and their ratio (NLR) in ST-segment elevation myocardial infarction (STEMI) and coronavirus disease (COVID)-19 and explore their associations with clinical events and structural damage. Circulating neutrophil, lymphocyte and NLR were sequentially measured in 659 patients admitted for STEMI and in 103 COVID-19 patients. The dynamics detected in STEMI (within a few hours) were replicated in COVID-19 (within a few days). In both entities patients with events and with severe structural damage displayed higher neutrophil and lower lymphocyte counts. In both scenarios, higher maximum neutrophil and lower minimum lymphocyte counts were associated with more events and more severe organ damage. NLR was higher in STEMI and COVID-19 patients with the worst clinical and structural outcomes. A canonical deregulation of the immune response occurs in STEMI and COVID-19 patients. Boosted circulating innate (neutrophilia) and depressed circulating adaptive immunity (lymphopenia) is associated with more events and severe organ damage. A greater understanding of these critical illnesses is pivotal to explore novel alternative therapies.

## 1. Introduction

ST-segment elevation MI (STEMI) constitutes the most critical presentation of ischemic heart disease. Although early reperfusion, mainly by percutaneous intervention, has exponentially increased acute survivorship, a significant number of patients still display cardiovascular adverse events (e.g., heart failure and/or death) during the following months and years [1]. The immune system actively participates in the repair process after STEMI by removing apoptotic cardiomyocytes and damaged extracellular matrix, subsequently paving the way to form a proper fibrotic scar [2]. However, an uncontrolled and exacerbated response during the acute inflammatory phase or a prolonged reactivity has been linked to detrimental effects [3,4,5].

Although ischemic heart disease, including STEMI as its most serious form, is (and probably will be) the main cause of death in the western world in the coming decades, coronavirus disease 2019 (COVID-19) currently represents the main global concern of health systems. In this setting, preliminary studies have also pointed to the negative effect of severe immune system deregulation on patient prognosis [6,7].

Composed by innate and adaptive responses, the immune system is designed to work in a complementary and cooperative way in infectious (such as COVID-19) and non-infectious (such as STEMI) critical diseases. However, in both scenarios, current clinical data suggest that excessively amplified innate and decreased adaptive immunity contribute to unnecessary structural damage and worse outcomes.

The specific aims of the present study were to (1) analyze the dynamics of circulating immune cells after STEMI and COVID-19, (2) investigate the relationship with the occurrence of adverse events, and (3) assess its association with structural damage evaluated by cardiac magnetic resonance (CMR) in STEMI patients and either chest X-ray or computed tomography (CT) in COVID-19 patients.

## 2. Materials and Methods

### 2.1. Ethics Statement

The study conformed to the principles for use of human subjects outlined in the Declaration of Helsinki. The study protocol was approved by the local Research Ethics Committee and written informed consent was obtained from all subjects (PE-BB-Covid19, PI08/0128, PI11/02323 and PIE15/00013).

### 2.2. Study in STEMI Patients

We prospectively included 730 consecutive patients admitted to a university hospital for a first STEMI between 2004 and 2019 treated with primary coronary intervention and undergoing pre-discharge CMR. Inclusion criteria were stable clinical course during admission, no contraindication to CMR, and no condition related to an alteration of the immune system apart from STEMI.

**Figure 1:**
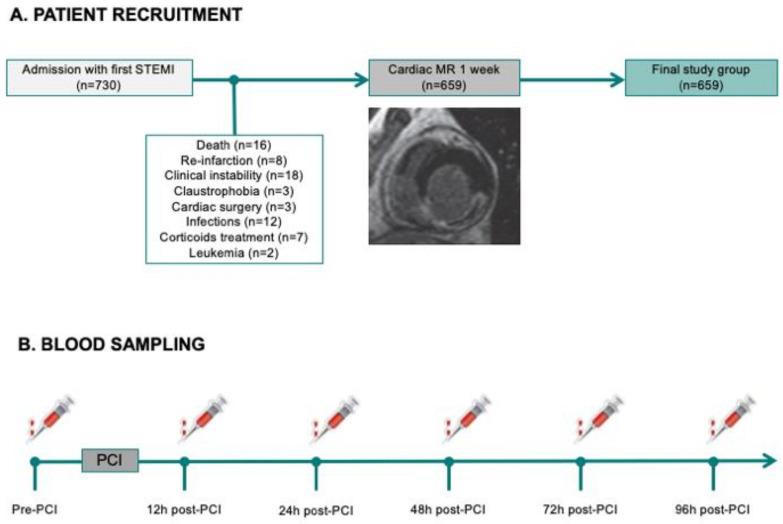
Flowchart of the STEMI study group, showing STEMI patient recruitment (**A**) and blood sampling (**B**). MR: magnetic resonance; PCI: primary coronary intervention; STEMI: ST-segment elevation myocardial infarction.

We excluded 71 patients for the following criteria: death (*n* = 16), re-infarction (*n* = 8), severe clinical instability precluding CMR (*n* = 18), claustrophobia (*n* = 5), cardiac surgery (*n* = 3), infections (*n* = 12), corticoids treatment (*n* = 7), and leukemia (*n* = 2). The final study group comprised 659 patients (Figure 1A).

#### 2.2.1. Circulating White Blood Cell Counts

Total leukocyte, neutrophil, monocyte, lymphocyte, and eosinophil (×1000 cells/mL) counts were measured upon patient arrival, and at 12, 24, 48, 72, and 96 h after revascularization using a commercial assay based on cytochemical light scatter and light absorption measurements (ADVIA^®^ 120 Hematology System from Siemens Diagnostics). Neutrophil-to-lymphocyte ratio (NLR) was also calculated (Figure 1B).

#### 2.2.2. CMR

Patients included in the study group were examined with a 1.5 T System (Sonata Magnetom, Siemens, Erlangen, Germany) 7 ± 2 days after STEMI following a previously validated study protocol [8,9]. All studies were performed by local cardiologists specialized in CMR with more than 10 years experience and quantified offline by two different operators with 3 years experience blinded to all patient data using customized software (QMASS MR, 6.1.5, Medis, Leiden, The Netherlands). Further details on the technical aspects of CMR acquisition, sequences, and quantification can be found in the Supplementary Materials.

Infarct size [% of left ventricular (LV) mass] was assessed as the percentage of LV mass showing late gadolinium enhancement [8,9,10].

**Figure 2:**
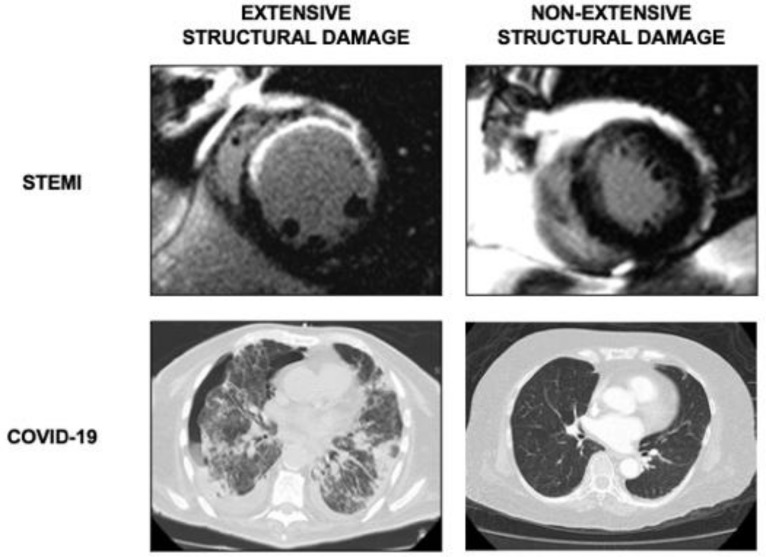
Extension of structural damage. In STEMI patients (upper panel), CMR images of extensive (left) and non-extensive (right) infarct size. In COVID-19 patients (lower panel), CT studies of bilateral (left) and unilateral (right) pneumonia. CMR: cardiac magnetic resonance. COVID-19: coronavirus disease 2019. CT: computed tomography. STEMI: ST-segment elevation myocardial infarction.

The patients were categorized according to infarct size: extensive if >30% of LV mass (Figure 2). This cut-off point has been previously validated for prediction of ventricular remodeling and adverse cardiovascular events [8].

#### 2.2.3. Follow Up

Major adverse cardiac events (MACE) consisted of death or heart failure, whichever occurred first. Current definitions were applied [11,12]. During a median follow-up of 5.2 years all MACE were periodically updated, and consensus among three cardiologists was required to assign a cardiac event.

### 2.3. Study in COVID-19 Patients

A total of 112 adult patients with severe acute respiratory syndrome-CoV2 infection confirmed by pharyngeal swab nucleic acid test, admitted to a university hospital between March and June 2020 were included in this study. Baseline characteristics and laboratory data were prospectively recorded. Patient inclusion was in accordance to the “Diagnosis and Treatment Protocol for COVID-19 (Trial Version 7)” distributed by the National Health Commission [13].

**Figure 3:**
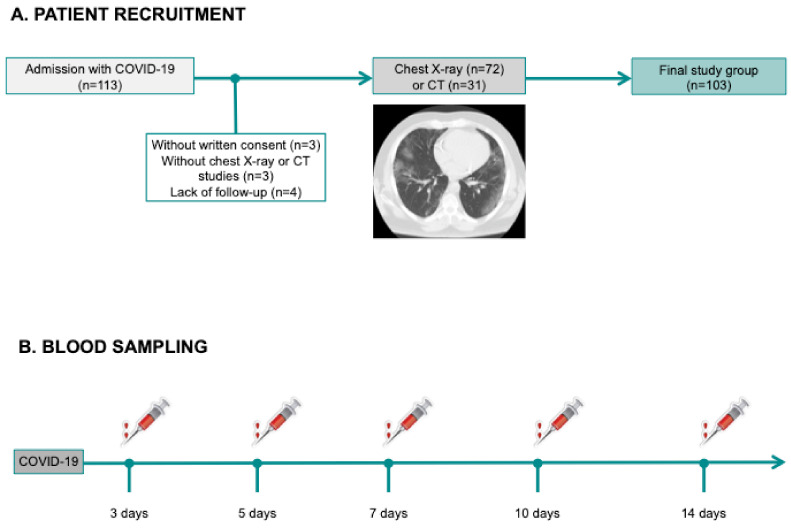
Flowchart of the COVID-19 study group, showing COVID-19 patient recruitment (**A**) and blood sampling (**B**). COVID-19: coronavirus disease 2019; CT: computed tomography.

Exclusion criteria were patients without written consent (*n* = 3), without imaging studies (*n* = 3), or lack of follow-up (*n* = 4), leaving a final study group of 103 patients (Figure 3A).

#### 2.3.1. Circulating White Blood Cell Counts

Total leukocyte, neutrophil, monocyte, lymphocyte, and eosinophil (×1000 cells/mL) counts were measured upon patient arrival, and at 3, 5, 7, 10 and 14 days by an automated blood cell counter (ADVIA^®^ 120 Hematology System from Siemens Diagnostics). As a proxy of the imbalance between innate immunity and adaptive immunity, we calculated NLR (Figure 3B). As there is no established cut-off value for NLR, in both f STEMI and COVID-19 populations, we applied the respective median values.

#### 2.3.2. Image Analysis

All patients underwent various imaging studies to monitor the course of COVID-19 following physician criteria and guidelines for diagnosis and treatment of the disease [13]. Patients were categorized according to presence of bilateral pneumonia during hospitalization in any of the imaging studies performed either by chest X-ray (*n* = 72) or CT (*n* = 31).

Chest CT (Aquilion, Toshiba Medical Systems, Tochigi, Japan) studies were performed using a 64-section scanner with the following parameters: tube voltage, 120 kV; automatic tube current (180–400 mA); 0.625 mm collimation, and 1.5 pitch. CT images were evaluated using a window level of 40 HU and width of 300 HU.

Presence of bilateral pneumonia as derived from detection of ground-glass opacities was used to define extensive structural damage in COVID-19 patients (Figure 2). Ground-glass opacification was defined as increased lung parenchymal attenuation that did not obscure the underlying vessels [14,15,16]. All the images were reviewed by two experienced chest radiologists and confirmed by the clinicians in charge of patients.

Even though bilateral pneumonia is considered the strong indicator of the severity of COVID-19 patients, we also classified patients according to “Radiographic Assessment of Lung Edema” (RALE) score [17]. A score from 0 to 8 was assessed to each patient, ranging from the absence of any pathological sign (score 0) to the complete pathological involvement of lung parenchyma (score 8). Afterwards, patients were divided into three groups as follows: (i) mild (score 0 to 2), (ii) moderate (score 3 to 5), and (iii) severe (score 6 to 8) to evaluate its association with the maximum neutrophil count, minimum lymphocyte count, and maximum NLR.

#### 2.3.3. Follow Up

Adverse events were defined as death and/or admission to intensive care unit (ICU) [6,7] and were registered during a median follow-up of 100 days. All data were revised by two physicians.

### 2.4. Statistical Analysis

Data were tested for normal distribution using the Kolmogorov–Smirnov test. Continuous normally distributed data were expressed as the mean ± the standard deviation of the mean and compared using the unpaired Student’s t-test. Non-parametric data were expressed as the median with the interquartile range and compared using the Mann–Whitney U-test. Group percentages were compared using the Chi-squared test or Fisher’s exact test where appropriate. For cell counts, we also assessed whether measurements were within the normal ranges based on previously established values by our laboratory. Two-sided *p*-values of less than 0.05 were considered statistically significant. All statistical tests were performed using SPSS 19.0 (SPSS, Inc., Chicago, IL, USA).

## 3. Results

In the STEMI cohort, the mean age was 60 ± 12 years, and 532 (81%) were male patients. Compared to STEMI, COVID-19 patients were significantly older (age: 69 ± 16 years, *p* < 0.001) with a lower male percentage (48%, *p* < 0.001). The number of smokers was higher in STEMI than in COVID-19 patients, whereas no differences were detected in other risk factors such as diabetes mellitus, hypertension, or hypercholesterolemia (Supplementary Table S1).

### 3.1. Dynamics of Neutrophil and Lymphocyte Counts and NLR in STEMI and COVID-19 Patients

Baseline and clinical characteristics of both (STEMI and COVID-19) groups are shown in Table 1 and Table 2.

**Figure 4:**
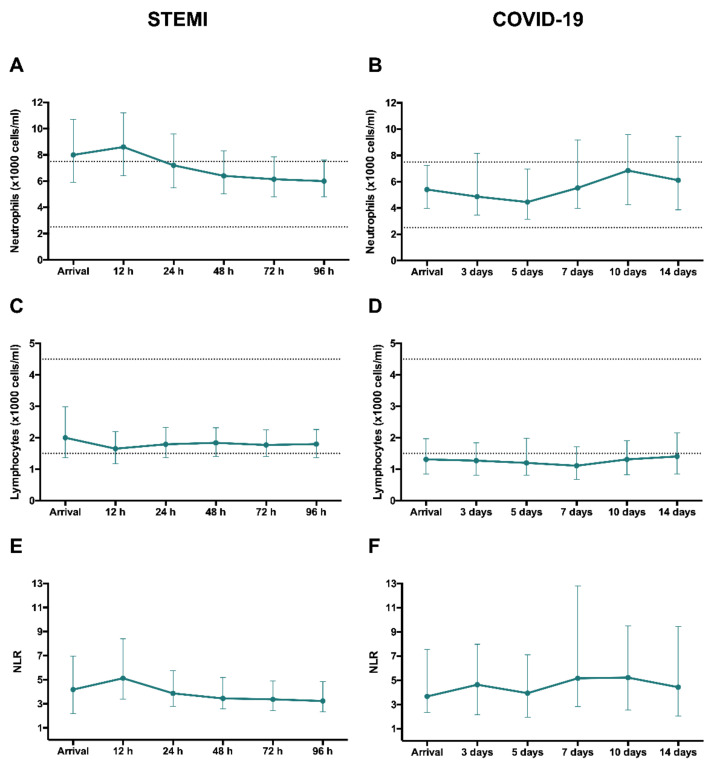
Evolution of neutrophil and lymphocyte counts and NLR after reperfused STEMI and COVID-19. Time course of median neutrophil and lymphocyte (×1000 cells/mL) counts and NLR in the entire cohort of STEMI patients (**A**,**C**,**E**, respectively) and COVID-19 (**B**,**D**,**F**, respectively). Data were expressed as median [quartile 25-quartile 75]. Dotted lines represent the upper and lower ranges of normality. COVID-19: coronavirus disease 2019; NLR: neutrophil-to-lymphocyte ratio; STEMI: ST-segment elevation myocardial infarction.

In STEMI patients, neutrophil count and NLR peaked in the first measurement (12 h) after revascularization (Figure 4A,E). A marked drop in circulating lymphocytes could be observed at that time point (Figure 4C).

In COVID-19 patients, the number of circulating neutrophils increased steadily from day 5 onward (Figure 4B). Lymphocyte counts were below the minimum adult normal range at all measurements (Figure 4D).

Thus, we observed a similar tendency in terms of neutrophilia, lymphopenia and increased NLR in STEMI and COVID-19 patients, though the latter displayed a more protracted time course regarding neutrophil count.

The dynamics of total leukocyte count, monocyte, and eosinophil counts are shown in Supplementary Figure S1. Again, STEMI and COVID-19 patients displayed similar patterns, characterized by monocytosis (a few days after admission) and eosinopenia (already detected in the first samples).

### 3.2. Association with Occurrence of Adverse Events and Resultant Structural Damage

#### 3.2.1. Adverse Events

In STEMI patients during a median follow-up of 5.2 years, there were 110 MACE (37 cardiac deaths, and 73 readmissions for heart failure). In patients with COVID-19 during a median follow-up of 100 days, 30 events (30%) were detected: 15 deaths and 15 admissions to ICU.

**Figure 5:**
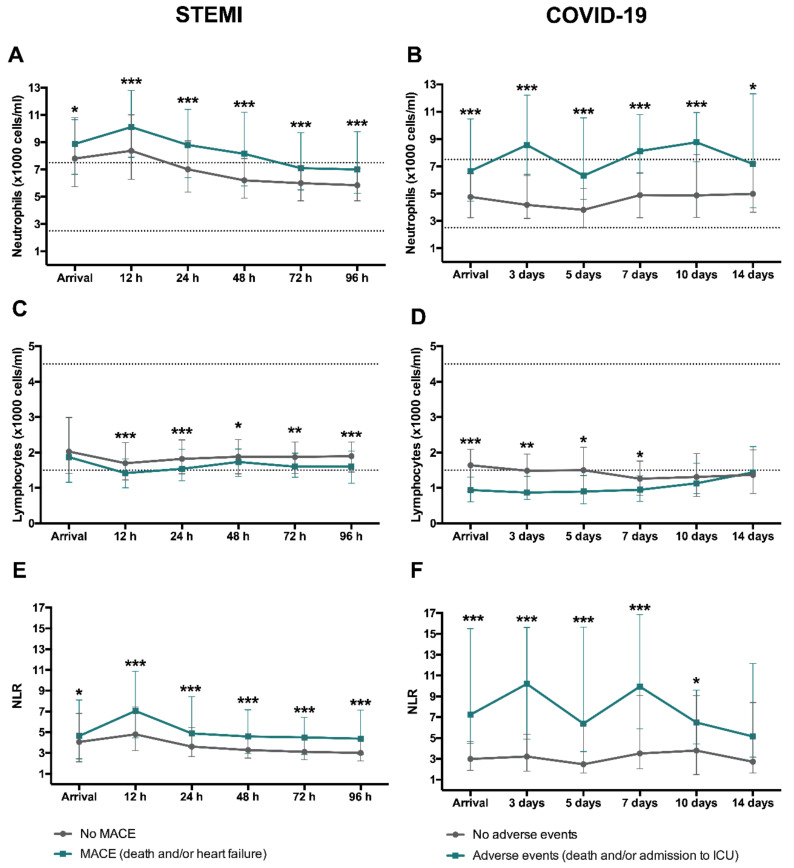
Time course of neutrophil and lymphocyte counts and NLR according to occurrence of adverse events. In STEMI (*n* = 659), MACE included death, and readmission for heart failure. Evolution of median neutrophil (**A**) and lymphocyte (**C**) (×1000 cells/mL) counts as well as NLR (**E**) according to MACE. In COVID-19 patients (*n* = 103), adverse events were defined as death and/or admission in ICU. Dynamics of median neutrophil (**B**) and lymphocyte (**D**) (×1000 cells/mL) counts as well as NLR (**F**) according to adverse events. Data were expressed as median [quartile 25-quartile 75]. Dotted lines represent the upper and lower ranges of normality. * *p* < 0.05, ** *p* < 0.01, *** *p* < 0.001. COVID-19: coronavirus disease 2019; ICU: intensive care unit; MACE: major adverse cardiac events; NLR: neutrophil-to-lymphocyte ratio; STEMI: ST-segment elevation myocardial infarction.

The baseline and clinical characteristics of STEMI and COVID-19 patients with and without adverse events are shown in Table 1 and Table 2, respectively. Figure 5 displays the association of neutrophil and lymphocyte counts and NLR with occurrence of adverse events.

After STEMI, patients with MACE had a significantly higher circulating neutrophil count and NLR upon arrival and at all measurements after revascularization, with a marked peak at 12 h after revascularization (Figure 5A,E). In contrast, patients with MACE displayed a reduced number of circulating lymphocytes post-reperfusion and this tendency persisted over the following days (Figure 5C).

In parallel with these results, COVID-19 patients showing adverse events presented higher circulating neutrophil (Figure 5B), lower lymphocyte count (Figure 5D), and heightened NLR (Figure 5F) at admission and at all subsequent measurements.

Circulating monocytosis (several days after admission) and eosinopenia (since arrival) occurred in STEMI and COVID-19 patients with events (Supplementary Figure S2).

#### 3.2.2. Structural Damage

**Figure 6:**
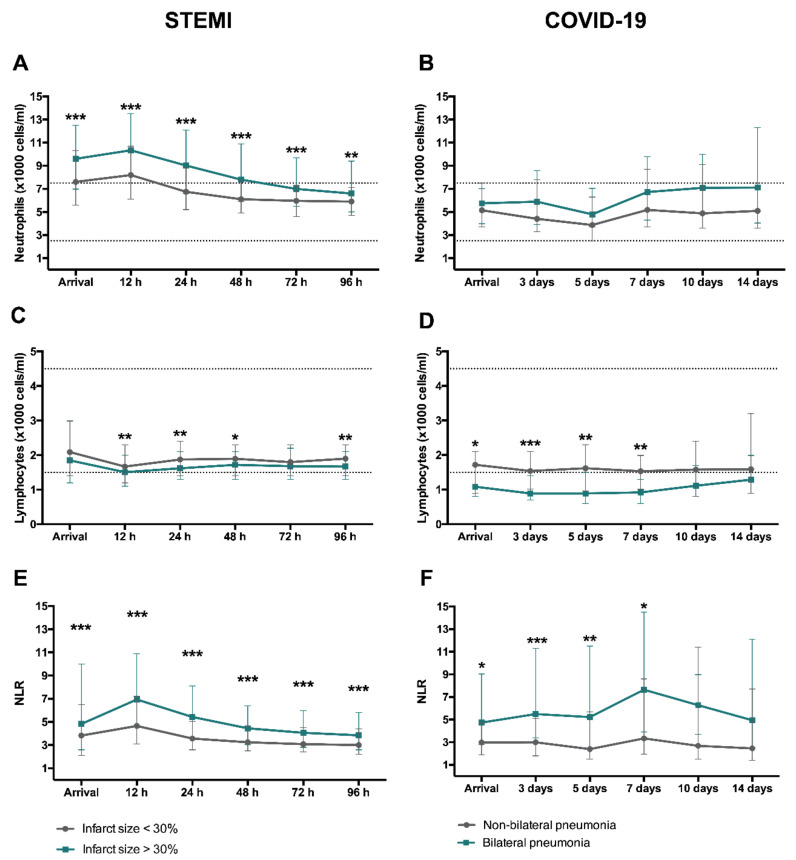
Dynamics of circulating neutrophil and lymphocyte counts and NLR according to structural damage after reperfused STEMI and COVID-19. In STEMI, patients were dichotomized according to the extension of CMR-derived infarct size (extensive: >30% of LV mass and non-extensive: <30% of LV mass). Time course of median neutrophil (**A**) and lymphocyte (**C**) (×1000 cells/mL) counts as well as NLR (**E**) depending on infarct size. In COVID-19, patients were dichotomized according to the extension of CT-derived bilateral pneumonia. Time course of median neutrophil (**B**) and lymphocyte (**D**) (×1000 cells/mL) counts as well as NLR (**F**) depending on the presence of bilateral pneumonia. Data were expressed as median [quartile 25-quartile 75]. Dotted lines represent the upper and lower ranges of normality. * *p* < 0.05, ** *p* < 0.01, *** *p* < 0.001. CMR: cardiac magnetic resonance; COVID-19: coronavirus disease 2019; CT: computed tomography; LV: left ventricular; NLR: neutrophil-to-lymphocyte ratio; STEMI: ST-segment elevation myocardial infarction.

Supplementary Tables S2 and S3 show the respective baseline characteristics of STEMI and COVID-19 patients according to extent of structural damage. Figure 6 reflects the dynamics of circulating neutrophil and lymphocyte counts as well as NLR.

Patients with more extensive structural damage (large infarct size in case of STEMI and bilateral pneumonia in COVID-19) showed a similar pattern. Higher circulating neutrophil and reduced lymphocyte counts as well as an increased NLR were associated with presence of CMR-derived extensive infarction in STEMI and bilateral pneumonia in COVID-19 patients (Figure 6).

Circulating monocytosis and eosinopenia (as shown in Figure S3) associated with presence of extensive structural damage as detected by CMR (in STEMI) and either chest X-ray or CT (in COVID-19).

**Figure 7:**
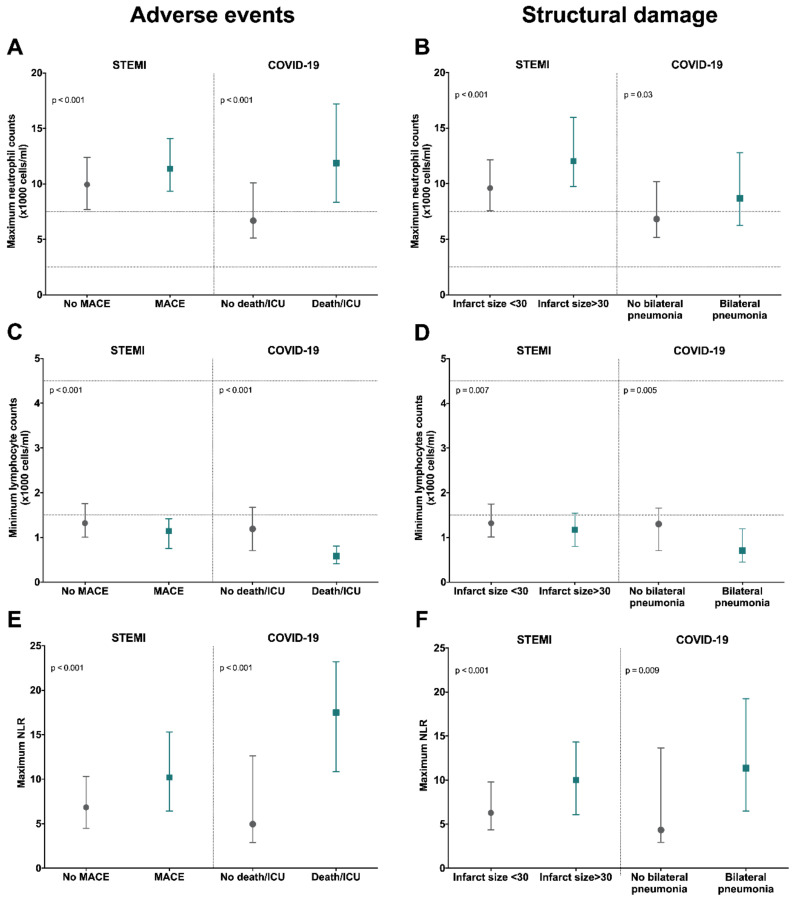
Association of maximum neutrophil and minimum lymphocyte count as well as maximum NLR with occurrence of adverse events and severe structural damage. Maximum neutrophil count (**A**,**B**), minimum lymphocyte count (**C**,**D**), and maximum NLR (**E**,**F**) as related to presence of adverse events (left panel) or severe structural damage (right panel). In STEMI patients, MACE included death and/or readmission for heart failure, whereas in the COVID-19 cohort, death and/or admission in ICU were regarded as adverse events. With respect to structural damage, STEMI patients were dichotomized according to CMR-derived infarct size and COVID-19 depending on the presence of bilateral pneumonia in either chest X-ray or CT images. Data were expressed as median [quartile 25-quartile 75]. Dotted lines represent the upper and lower ranges of normality. CMR: cardiac magnetic resonance; COVID-19: coronavirus disease 2019; CT: computed tomography; ICU: intensive care unit; MACE: major adverse cardiac events; NLR: neutrophil-to-lymphocyte ratio; STEMI: ST-segment elevation myocardial infarction.

**Figure 8:**
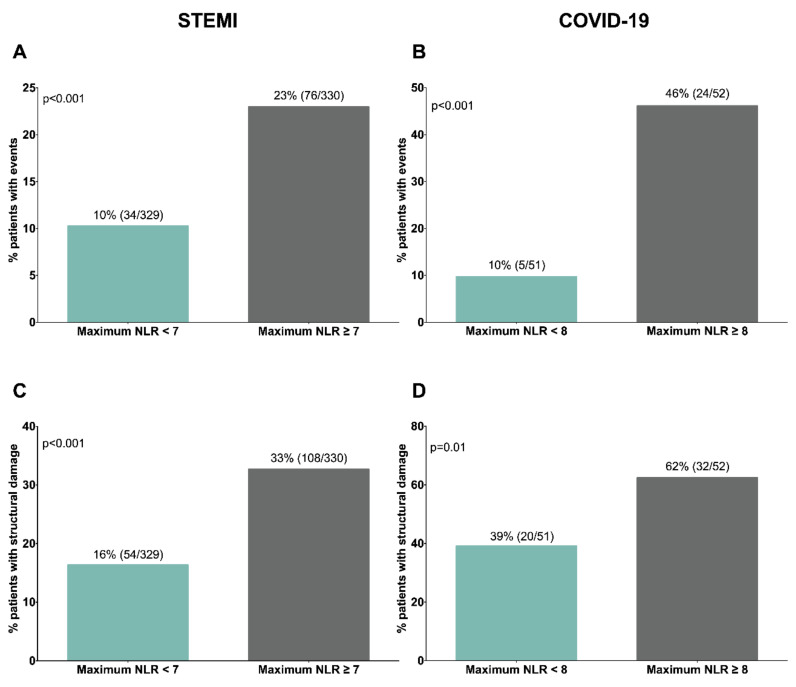
Percentage of patients experiencing adverse events or severe structural damage according to maximum NLR in STEMI and COVID-19 cohorts. Both populations were dichotomized according to the median value of maximum NLR (STEMI: 7 and COVID-19: 8). Percentage of patients showing adverse events: (**A**) STEMI: death and/or heart failure; (**B**) COVID-19: death and/or admission into ICU) or severe structural damage; (**C**) STEMI: CMR-derived infarct size >30% of left ventricular mass; (**D**) COVID-19: CT-derived bilateral pneumonia) according to maximum NLR. CMR: cardiac magnetic resonance; COVID-19: coronavirus disease 2019; CT: computed tomography; ICU: intensive care unit; NLR: neutrophil-to-lymphocyte ratio; STEMI: ST-segment elevation myocardial infarction.

To further illustrate these observations, we adopted dichotomic approaches (Figure 7). Higher maximum neutrophil count and NLR and lower minimum lymphocyte were detected in the groups of STEMI and COVID-19 patients with adverse events and extensive structural damage (bilateral pneumonia). Indeed, when structural damage was evaluated following RALE score, we observed that the higher the score is the more elevated maximum neutrophil counts and maximum NLR as well as more reduced minimum lymphocyte counts is (Supplementary Table S4). Using NLR as a proxy of the imbalance of innate over adaptive immunity, patients with augmented NLR (>median) displayed a significantly higher risk of adverse events and extensive structural damage (Figure 8).

## 4. Discussion

The key finding of this study is that a canonical inflammatory response occurs in both reperfused STEMI and COVID-19 patients. An excessive circulating innate and depressed circulating adaptive reaction is associated with poorer outcomes and more severe structural damage in target tissue (heart and lungs) as derived from gold standard imaging techniques. Investigating shared immune response mechanisms that play a role in the pathophysiology of these critical illnesses would be useful.

Ischemic heart disease, including STEMI as its most critical presentation, is, and probably will be, the main cause of death in western countries, but the outbreak of COVID-19 in December 2019 has brought a new threat to global healthcare systems. Although there are some differences between the two entities in baseline characteristics, preliminary results have indicated that the dynamics of circulating inflammatory cells in patients with severe lung damage and complicated clinical course mirror [6,7] those already reported in patients with large infarctions [5,18,19] and other critical infectious and non-infectious situations. This highlights a need to scrutinize the canonical time course of the main immune cell counts occurring in STEMI and COVID-19 patients, as well as to explore its association with adverse events and severe structural damage as evaluated using gold-standard imaging techniques.

### 4.1. Activation of Innate Immunity after STEMI and COVID-19

Although the role of neutrophils after reperfused STEMI has been widely addressed [5,20,21], similarities between the course of the innate immune response in STEMI and COVID-19 patients have not as yet been explored.

Based on our results, circulating neutrophil counts peak soon (a few hours) after revascularization in STEMI whereas a certain delay (up to several days) takes place before the same observation can be made in COVID-19 patients. Augmented counts, as a proxy of the predominance of uncontrolled circulating innate immunity, associates with worse clinical course and larger infarctions in STEMI. In the case of COVID-19, it relates to more severe lung damage, need of ICU, and death. In line with these results, further bench and bedside research has also suggested that neutrophilia is negatively correlated with systolic function, microvascular obstruction and adverse remodeling after STEMI [5,21,22].

Neutrophils, the most abundant leukocytes in peripheral blood, play a crucial role in heightening the inflammatory response, either by secreting toxic substances or by calling on more pro-inflammatory cells [23,24]. Following capture of sterile antigens (necrotic cardiomyocytes and damaged fragments from ischemic myocardium) in STEMI and viruses after COVID-19 principally by monocytes and dendritic cells, there is massive secretion of proinflammatory molecules (e.g., interleukin-1, interleukin-6, tumor necrosis factor-α, among others). In response to this strong chemotactic gradient, neutrophils seem to be activated and attracted to the injured tissue. Briefly, in response to potent inflammatory stimulus, distinct sets of adhesion molecules promote neutrophil rolling and attachment to post-capillary venules and entry into tissues. For neutrophils, firm adhesion requires activation of integrins and binding to intercellular adhesion molecule-1. Transendothelial migration follows, leading to direct injury on parenchymal cells through release of proteolytic enzymes to promote tissue cleaning [24,25]. Even though circulating neutrophils peaks at 12 h after coronary reperfusion, myocardial presence peaks at day 1 and gradually returns to baseline levels by day 7 [2].

Within the first hours and days, uncontrolled innate immune response initiates (and may also magnify) not only tissue (heart and lung) but also microvascular injury. As a consequence of this inflammatory milieu underlying STEMI and COVID-19, neutrophils adhere to capillary endothelium together with decondensed chromatin and antimicrobial proteins such as elastase and cathepsin G, causing formation of plugs in microvessels. Neutrophil plugging traps and immobilizes red blood cells as well as overactivated platelets, thus contributing to vein thrombosis, microvascular obstruction and exacerbated endothelium damage [26,27]. In fact, clinical and experimental studies in both entities have suggested a negative impact of microvascular injury due to neutrophil plugging [28,29].

Collectively, the positive association detected between augmented circulating neutrophil counts and severe structural damage and poorer outcomes in these entities could be attributed to the disproportionate inflammatory response itself in the target tissue and also to the formation of intravascular plugs which enhance microvascular injury. However, a recent meta-analysis reported that superadded bacterial infections rate is higher in those COVID-19 patients requiring ICU [30]. Therefore, neutrophilia detected in this subgroup of patients might be also due to a higher rate of bacterial co-infections.

Apart from neutrophils, eosinophils also participate in innate immunity and are notably altered in some inflammatory scenarios (e.g., parasitic infection, asthma, and allergic reactions) [31]. Eosinophils were seen to decline dramatically after reperfusion and this drop was more pronounced in patients displaying worse outcomes and extensive structural damage in STEMI and COVID-19 patients. A previous study concluded that this early fall could be caused by their migration into the infarcted heart, as reflected by their presence in myocardial samples isolated from swine and patients [18].

Lastly, monocytes act as a bridge between innate and adaptive immunity. In our cohorts, monocyte counts steadily increased upon arrival and circulating monocytosis were strongly related to the resultant tissue structure and incidence of outcomes. Briefly, these cells are crucial for removing apoptotic cells in sterile inflammation (such as STEMI) as well as exogenous threats (in the case of COVID-19), paving the way for tissue repair and healing [2,32]. As a consequence, augmented circulating monocyte counts can unnecessarily expand tissue damage by triggering an excessive inflammatory response.

### 4.2. Implication of Adaptive Immunity after STEMI and COVID-19

Unlike its innate counterpart, adaptive immunity is crucial for attenuating inflammatory response. Lymphocytes, composed of a wide range of cellular phenotypes, can be considered truly strategic for the immune system since they regulate key steps to achieve a sustained response. In fact, under stress situations (e.g., trauma and sepsis), the acute inflammatory phase followed by activation of lymphopenia-mediated anti-inflammatory mechanisms has been widely demonstrated [33]. In line with these results, in our prospective registry of STEMI patients, a rapid drop in circulating lymphocytes after ischemia onset followed by a slow recovery after coronary reperfusion was detected, whereas an acute circulating lymphopenia was observed at all measurements after COVID-19.

Life-threatening situations such as major burns or sepsis relate to a significant decrease in CD4 cells, and patient survival goes along with lymphocyte count recovery. Similarly, in our study, we observed that lower peripheral lymphocyte counts were strongly associated with worse prognosis and extensive structural damage: large CMR-derived infarctions after STEMI and bilateral pneumonia in COVID-19 patients evaluated by either chest X-ray or CT studies. Collectively, circulating lymphopenia (as a proxy of diminished adaptive immunity) is associated with poorer clinical and structural outcomes.

Although the mechanisms underlying circulating lymphopenia are beyond the scope of our manuscript, the decline of peripheral lymphocytes in patients after both STEMI and COVID-19 could be explained by increased lymphocyte apoptosis [19,34] as well as their recruitment to the infarcted heart [19,35], lung interstitium [36], and mediastinal lymph nodes [37].

Activation of molecular pathways implicated in lymphocyte apoptosis have been also explored in this context. Immune checkpoints, and specifically programmed death (PD)-1, are negative co-stimulatory molecules which block the normal lymphocyte activation through T-cell receptor and major histocompatibility complex [38]. In clinical and experimental STEMI models, PD-1 expression in mononuclear leukocytes has been demonstrated to increase during ischemia and is followed by a sudden drop a few hours after reperfusion. The more pronounced the reduction in PD-1 expression, the more extensive the infarct size as derived from CMR [39]. Therefore, exacerbated expression of PD-1 axis during acute ischemia might contribute to the massive lymphocyte loss via apoptosis observed soon after reperfusion.

Despite the lack of results from exploring lymphopenia in COVID-19 patients, preliminary results in China also singled out immune checkpoints as master regulators of the fall in lymphocyte counts. An augmented expression of PD-1 was recently reported, and was greater in patients requiring ICU admission [40]. Consequently, and in line with STEMI patients, immune checkpoint upregulation might exert a central role in diminished lymphocyte survivorship, which could subsequently lead to a hampered specific defense against viral agents, uncontrolled innate immunity and more severe pulmonary damage.

### 4.3. Potential Therapeutic Implications

As previously mentioned, the immune system is a complex network involving various synergistic and antagonistic roles, classified into the innate and adaptive components. They should work in a complementary and cooperative way for an intact and fully effective response. Based on our data, patients with more events and severe organ damage display elevated NLR, indicating a disproportionate predominance of circulating innate (neutrophilia) over adaptive (lymphopenia) immunity. Therefore, therapeutic options aimed at preventing the severe depression of adaptive immunity and/or reducing the massive activation of the innate response could be explored in both entities to increase survivorship. In fact, several clinical trials have recently been published demonstrating the effects of anti-inflammatory drugs in the context of severe STEMI and COVID-19. Regarding STEMI, the Colchicine Cardiovascular Outcomes Trial (COLCOT) [41], and Low Dose Colchicine after Myocardial Infarction (LoDoCo-MI) [42] trials verified that treatment with low-dose colchicine is beneficial in terms of prevention and prognosis. In the case of COVID-19, the Randomized Evaluation of COVID-19 Therapy (RECOVERY) [43] trial also resulted in lower 28-day mortality in patients receiving corticoids. Therefore, these preliminary data indicate that interfering in the inflammatory cascade might be useful for enhancing prognosis in these two demanding entities. Concretely in our cohort, we observed that 57% of patients displaying adverse events (ICU and/or death) received corticosteroids treatment, which might interfere in the immune response as well as in the leukocyte counts. However, in the subgroup of COVID-19 patients who are not taking steroids, higher NLR was also observed to be associated with adverse events.

### 4.4. Limitations of the Study

Our study is limited by its observational nature; further mechanistic approaches are needed to characterize the role of white blood cell subtypes in target and non-target organ failure. Additionally, the sample size of confirmed COVID-19 patients count is relatively small and made up of patients admitted to hospital, meaning that the immune response at the very first stages of the disease was missed. As circulating procalcitonin levels were not determined in the entire cohort, this biomarker was not included in the table of COVID-19 patients’ baseline characteristics. Since COVID-19 patients requiring ICU displayed a higher rate of bacterial co-infections [30] and corticosteroids, these might be cofounding factors in the interpretation of neutrophilia and NLR in this cohort. Finally, the mechanisms involved in the changes of circulating white blood cells are beyond the scope of our study, whose objective was to describe and compare the dynamics of circulating leukocyte subtypes in the two entities.

## 5. Conclusions

The main representatives of innate and adaptive immunity follow a canonical course in STEMI and COVID-19 patients. Exacerbated circulating innate (neutrophilia and augmented NLR) and decreased adaptive (lymphopenia) immune response correlate with extensive structural damage as evidenced by gold-standard imaging techniques, and poor clinical outcomes. The presented results strongly suggest that better understanding of the pathophysiology of these devastating, critical illnesses is essential to explore new therapeutic opportunities with potential impact on survivorship.

## Figures and Tables

**Table 1:** Baseline characteristics of STEMI patients with and without MACE.

	All	Without MACE	With MACE	*p*-Value
**Patients, n**	659	549	110	
**Age (years)**	60 ± 12	58 ± 12	66 ± 12	<0.001
**Male sex, n (%)**	532 (81)	455 (83)	77 (70)	0.002
**Diabetes mellitus, n (%)**	146 (22)	115 (21)	31 (28)	0.1
**Hypertension, n (%)**	324 (49)	252 (46)	72 (65)	<0.001
**Hypercholesterolemia, n (%)**	302 (46)	250 (46)	52 (47)	0.7
**Smoker, n (%)**	371 (56)	317 (58)	54 (49)	0.09
**Heart rate (beats per minute)**	78 ± 19	77 ± 19	84 ± 21	<0.001
**Systolic blood pressure (mmHg)**	130 ± 30	131 ± 29	129 ± 33	0.6
**Creatinine (mg/dL)**	0.92 (0.8–1.1)	0.91 (0.8–1.09)	0.96 (0.8–1.18)	0.1
**Glucose (mg/dL)**	130 (107–167)	128 (105–161)	148 (119–188)	<0.001
**Killip class >1 (%)**				<0.001
**1**	549 (83)	475 (87)	74 (67)	
**>1**	110 (17)	74 (13)	36 (33)	
**Time from chest pain to first medical contact (min)**	190 (130–300)	180 (120–297)	225 (160–420)	0.002
**CK-MB mass peak value (ng/mL)**	164 (60–290)	151 (57–278)	196 (69–318)	0.06
**Anterior infarction, n (%)**	332 (50)	260 (47)	72 (65)	0.001
**TIMI flow grade before PCI (%)**				0.9
**0**	345 (52)	286 (52)	59 (54)	
**1**	44 (7)	36 (7)	8 (7)	
**2**	73 (11)	63 (11)	10 (9)	
**3**	197 (30)	164 (30)	33 (30)	
**TIMI flow grade after PCI (%)**				0.1
**0**	17 (3)	12 (2)	5 (4)	
**1**	6 (1)	4 (1)	2 (2)	
**2**	50 (7)	37 (7)	13 (12)	
**3**	586 (89)	496 (90)	90 (82)	
**Grace Risk Score**	135 ± 32	131 ± 29	160 ± 33	<0.001
**Timi Risk Score**	2 (1–4)	2 (1–4)	4 (2–6)	<0.001

Abbreviations: CK-MB: creatine kinase myocardial band; MACE: major adverse cardiac events; STEMI: ST-segment elevation myocardial infarction; PCI: primary coronary intervention; TIMI: thrombolysis in myocardial infarction.

**Table 2:** Baseline characteristics of COVID-19 patients with and without adverse events (death/ICU admission).

	All	No Death/ICU	Death/ICU	*p*-Value
**Patients, n**	103	73	30	
**Age (years)**	69 ± 16	69 ± 17	69 ± 10	0.9
**Male sex, n (%)**	49 (48)	27 (37)	22 (73)	<0.001
**Diabetes mellitus, n (%)**	25 (24)	15 (21)	10 (33)	0.1
**Hypertension, n (%)**	57 (55)	39 (53)	18 (60)	0.4
**Hypercholesterolemia, n (%)**	42 (41)	29 (40)	13 (43)	0.6
**Smoker, n (%)**	5 (5)	5 (7)	0 (0)	0.1
**Heart rate (beats per minute)**	89 ± 18	88 ± 18	93 ± 20	0.2
**Systolic blood pressure (mmHg)**	131 ± 26	131 ± 26	133 ± 26	0.7
**Time to symptoms to first medical contact (min)**	5 (1–10)	5 (0–11)	6 (3–10)	0.7
**Creatinine (mg/dL)**	0.86 (0.74–1.11)	0.85 (0.69–1.09)	0.95 (0.83–1.38)	0.06
**Glucose (mg/dL)**	114 (97–135)	110 (96–126)	129 (112–168)	0.002
**Previous cardiovascular disease (%)**	24 (23)	16 (22)	8 (27)	0.5
**COPD (%)**	6 (6)	4 (5)	2 (7)	0.8
**D-dimer (ng/mL)**	602 (297–1248)	379 (234–760)	1125 (635–3210)	<0.001
**GOT (U/L)**	35 (23–52)	31 (20–45)	47 (37–67)	<0.001
**GPT (U/L)**	23 (14–44)	19 (12–33)	32 (23–61)	0.002
**LDH (U/L)**	590 ± 243	500 ± 202	793 ± 209	<0.001
**CRP (mg/L)**	45 (10–88)	33 (5–61)	89 (54–210)	<0.001
**Hydroxychloroquine (%)**	72 (70)	44 (60)	28 (93)	0.003
**Azithromycin (%)**	63 (61)	38 (52)	25 (83)	0.004
**Tocilizumab (%)**	31 (30)	11 (15)	20 (67)	<0.001
**Corticoids (%)**	27 (26)	10 (14)	17 (57)	<0.001

Abbreviations: COPD: chronic obstructive pulmonary disease; COVID: coronavirus disease; CRP: C-reactive protein; GOT: glutamic oxaloacetic transaminase; GPT: glutamate-pyruvate transaminase; ICU: intensive care unit; LDH: lactate dehydrogenase.

## Supplementary Materials

The following are available online at https://www.mdpi.com/2077-0383/9/11/3484/s1. Figure S1: Evolution of total leukocyte, monocyte, and eosinophil counts after reperfused STEMI and COVID. Figure S2. Time-course of total leukocyte, monocyte, and eosinophil counts according to the occurrence of adverse events. Figure S3. Dynamics of total leukocyte, monocyte, and eosinophil counts according to structural damage after reperfused STEMI and COVID-19. Table S1. Baseline characteristics of STEMI and COVID-19 patients. Table S2. Baseline characteristics of STEMI patients with extensive and non-extensive infarction. Table S3. Baseline characteristics of COVID-19 patients with bilateral pneumonia and without bilateral pneumonia. Table S4. Association between RALE score and the maximum neutrophil count, minimum lymphocyte count and maximum NLR in COVID-19 patients. References [44,45] are cited in the supplementary materials.
