# Supplementary material for: Similar Clinical Course and Significance of Circulating Innate and Adaptive Immune Cell Counts in STEMI and COVID-19
Source: J Clin Med. 2020 Oct 28;9(11):3484. doi: 10.3390/jcm9113484 (PMC7692467; doi:10.3390/jcm9113484)
Supplement: Supplementary file 1 [file jcm-09-03484-s001.pdf]

SUPPLEMENTARY MATERIAL

A. Material and methods

Study in ST-segment elevation myocardial infarction (STEMI)-patients

- Cardiovascular magnetic resonance acquisition, sequences, and quantification

Images were acquired by a phased-array body surface coil during breath-holds and were triggered by electrocardiography. All studies were performed by local cardiologists specialized in cardiac magnetic resonance (CMR) with more than 10 years experience and quantified offline by two different operators with 3 years experience blinded to all patient data using customized software (QMASS MR, 6.1.5, Medis, Leiden, The Netherlands). Traditional CMR data were prospectively recorded and immediately included in the registry database [1,2].

Cine images were acquired in two-, three-, and four-chamber views, and in short-axis views using a steady state free precession sequence (repetition time/echo time: 2.8/1.2 ms; flip angle: 58 degrees; matrix: 256 × 300; field of view: 320 × 270 mm; slice thickness: 7 mm) [1,2].

Late gadolinium enhancement imaging was performed 10 to 15 minutes after administering 0.1 mmol/kg of gadolinium diethylenetriaminepentaacetic acid (Magnograf, Juste S.A.Q.F., Madrid, Spain) in the same locations as in the cine images using a segmented inversion recovery steady state free precession sequence (repetition time/echo time: 750/1.26 ms; flip angle: 45 degrees; matrix: 256 × 184; field of view: 340 × 235 mm; slice thickness: 7 mm). Inversion time was adjusted to nullify normal myocardium [1,2].

Black blood, T2-weighted short TI inversion recovery sequences in the same short-axis view as the cine sequences were carried out all in mid-diastole. A half-Fourier acquisition single-shot turbo spin echo multisection sequence was used (recovery time: two R-R intervals; echo time: 33 ms; inversion time: 170 ms; slice thickness: 8 mm; interslice interval: 2 mm; flip angle: 160 degrees; matrix: 256 × 151; bandwidth: 781 Hz/pixel). Additionally, a segmented turbo-spin echo sequence was obtained with one slice per breath-hold (recovery time: two R-R intervals; echo time: 100 ms; inversion time: 170 ms; slice thickness: 8 mm; interslice interval: 2 mm; flip angle: 180 degrees; matrix: 256 × 146; bandwidth: 235 Hz/pixel) [1,2].

B. Figures

SUPPLEMENTARY FIGURE 1

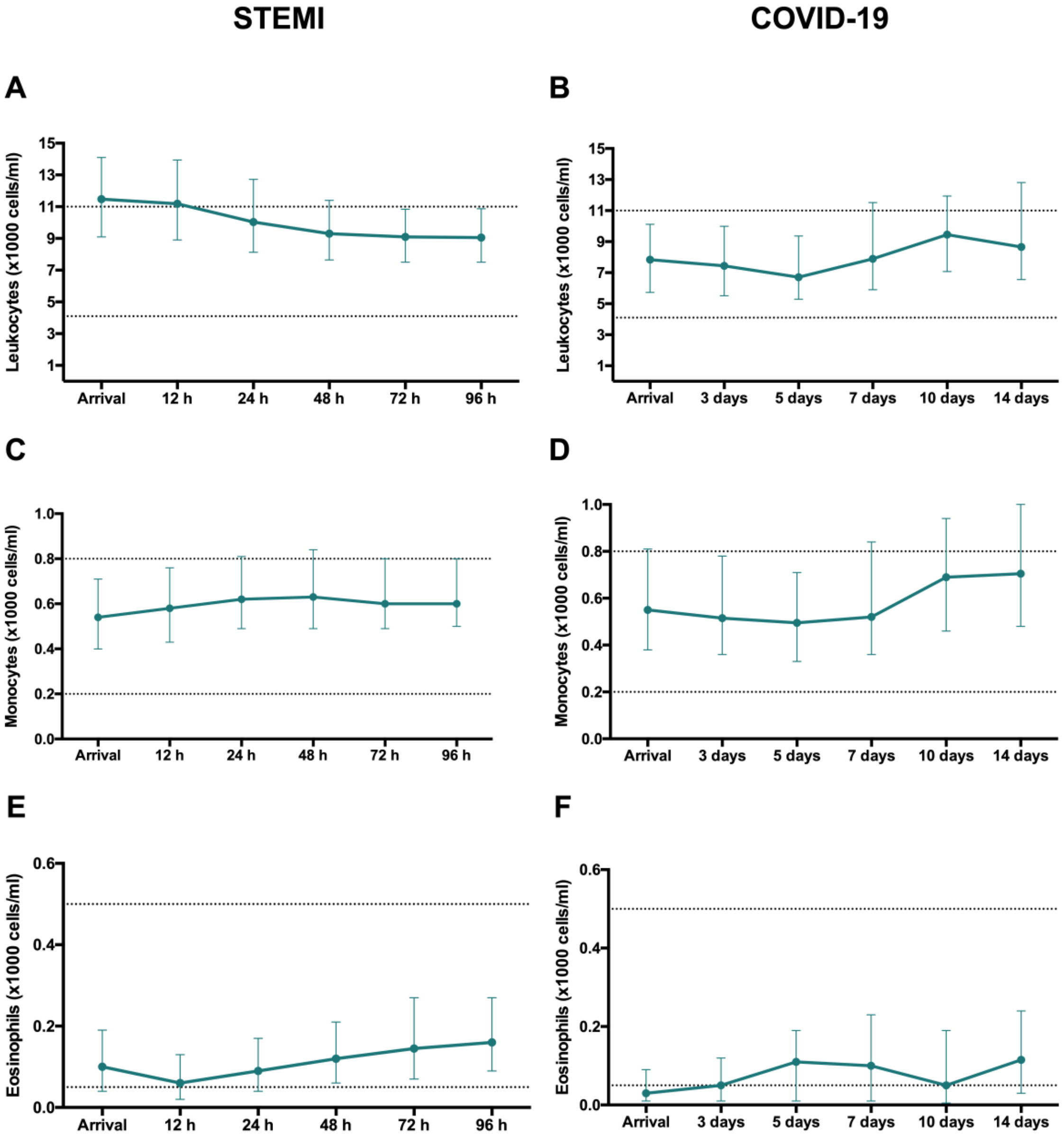

Figure S1. Evolution of total leukocyte, monocyte, and eosinophil counts after reperfused STEMI and COVID-19. Temporal evolution of median total leukocyte, monocytes and eosinophils (×1,000 cells/ml) counts in the whole study group of STEMI patients (a, c, and e respectively) and COVID-19 (b, d and f, respectively). Data were expressed as median [quartile 25-quartile 75]. Dotted lines represent the upper and lower ranges of normality. COVID-19: coronavirus disease 2019; STEMI: ST-segment elevation myocardial infarction.

SUPPLEMENTARY FIGURE 2

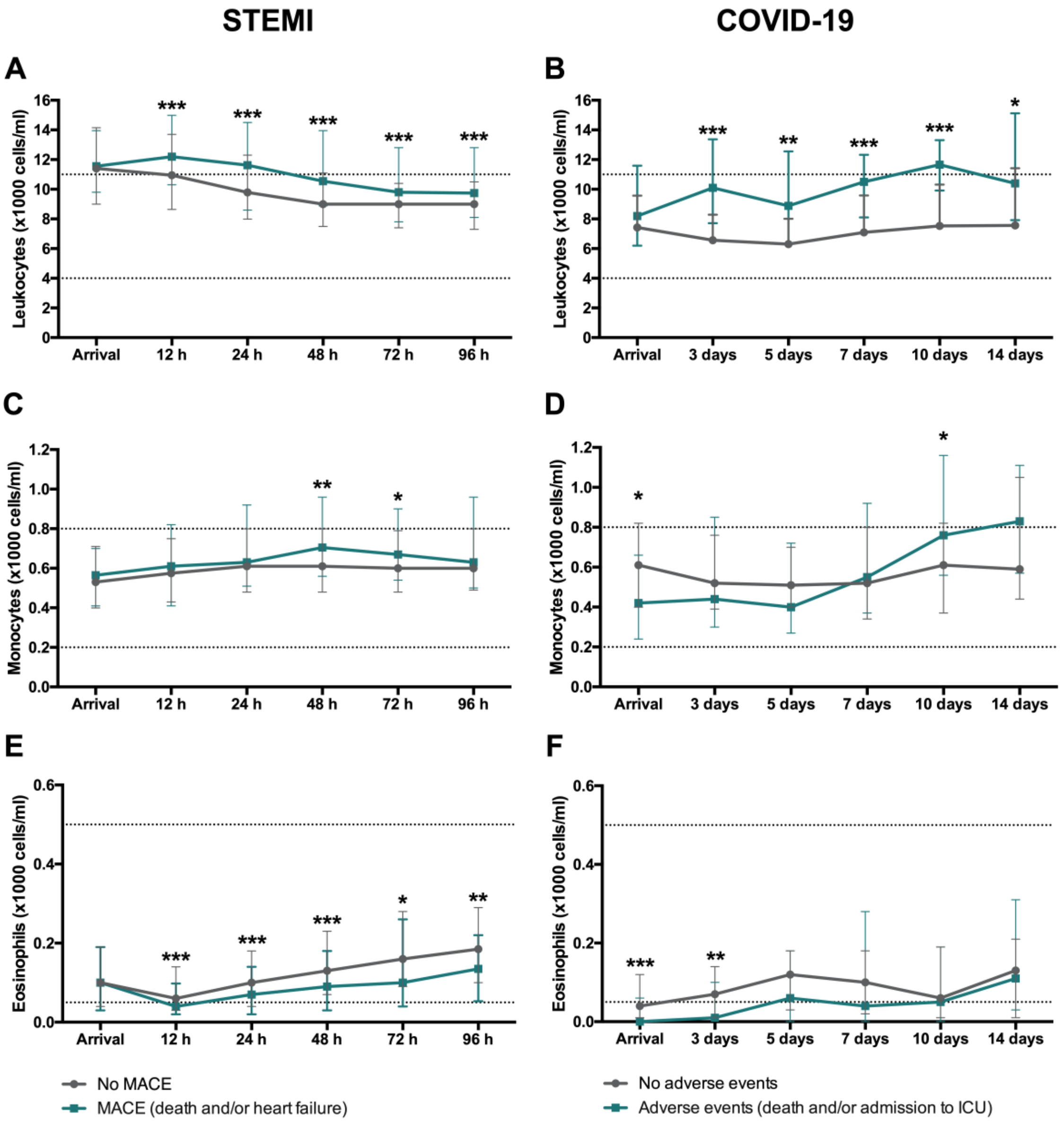

Figure S2. Time-course of total leukocyte, monocyte, and eosinophil counts according to the occurrence of adverse events. In STEMI (n=659), MACE included death and readmission for heart failure. Evolution of median total leukocyte (a), monocyte (c) and eosinophil (e) (×1,000 cells/ml) counts according to MACE. In COVID-19 patients (n=103), adverse events were defined as death and/or admission in ICU. Dynamics of median total leukocytes (b), monocytes (d) and eosinophils (f) (×1,000 cells/ml) according to adverse events. Data were expressed as median [quartile 25-quartile 75]. Dotted lines represent the upper and lower ranges of normality. *P<0.05, **P<0.01, ***P<0.001. COVID-19: coronavirus disease 2019; ICU: intensive care unit; MACE: major adverse cardiac events; STEMI: ST-segment elevation myocardial infarction.

SUPPLEMENTARY FIGURE 3

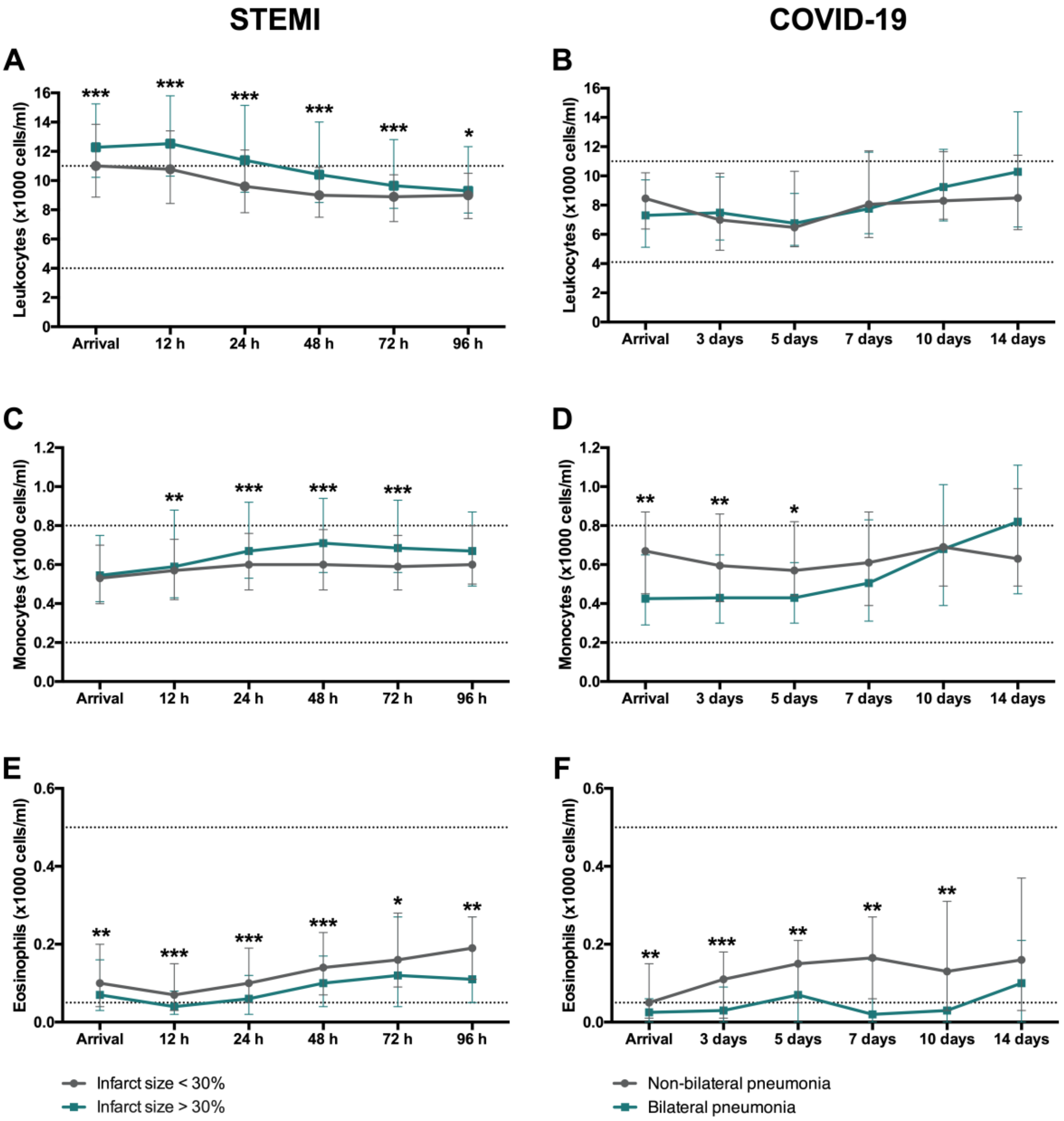

Figure S3. Dynamics of total leukocyte, monocyte, and eosinophil counts according to structural damage after reperfused STEMI and COVID-19. In STEMI, patients were dichotomized according to the extension of CMR-derived infarct size (extensive: >30% of LV mass and non-extensive: <30% of LV mass). Time-course of median leukocyte (a), monocyte (c), and eosinophil (e) (×1,000 cells/ml) counts depending on infarct size. In COVID-19, patients were dichotomized according to the extension of CT-derived bilateral pneumonia. Time course of median leukocyte (b), monocyte (d), and eosinophil (f) (×1,000 cells/ml) counts depending on the presence of bilateral pneumonia. Data were expressed as median [quartile 25-quartile 75]. Dotted lines represent the upper and lower ranges of normality. *P<0.05, **P<0.01, ***P<0.001. CMR: cardiac magnetic resonance; COVID-19: coronavirus disease 2019; CT: computed tomography; LV: left ventricular; NLR: neutrophil-to-lymphocyte ratio; STEMI: ST-segment elevation myocardial infarction.

C. Tables

Table S1. Baseline characteristics of STEMI and COVID-19 patients.

	STEMI	COVID-19	*p*-value
**Patients, n**	659	103	
**Age (years)**	60±12	69±16	<0.001
**Male sex, n (%)**	532 (81)	49 (48)	<0.001
**Diabetes mellitus, n (%)**	146 (22)	25 (24)	0.6
**Hypertension, n (%)**	324 (49)	57 (55)	0.2
**Hypercholesterolemia, n (%)**	302 (46)	42 (41)	0.3
**Smoker, n (%)**	371 (56)	5 (5)	<0.001

Abbreviations: COVID-19: Coronavirus disease 2019. STEMI: ST-segment elevation myocardial infarction.

Table S2. Baseline characteristics of STEMI patients with extensive and non-extensive infarction.

	All	Infarct size<30%	Infarct size>30%	*p*-value
**Patients, n**	659	497	162	
**Age (years)**	60±12	60±12	59±13	0.5
**Male sex, n (%)**	532 (81)	401 (81)	131 (81)	0.9
**Diabetes mellitus, n (%)**	146 (22)	98 (20)	48 (30)	0.008
**Hypertension, n (%)**	324 (49)	247 (50)	77 (48)	0.6
**Hypercholesterolemia, n (%)**	302 (46)	222 (45)	80 (49)	0.3
**Smoker, n (%)**	371 (56)	277 (56)	94 (58)	0.6
**Heart rate (beats per minute)**	78±19	76±18	86±20	<0.001
**Systolic blood pressure (mmHg)**	130±30	132±31	126±29	0.06
**Creatinine (mg/dL)**	0.92 (0.8-1.1)	0.91 (0.8-1.1)	0.94 (0.83-1.1)	0.1
**Glucose (mg/dL)**	130 (107-167)	128 (105-161)	138 (114-177)	0.009
**Killip class >1 (%)**				<0.001
**1**	549 (83)	439 (88)	110 (68)	
**>1**	110 (17)	58 (12)	52 (32)	
**Time from chest pain to first medical contact (min)**	190 (130-300)	181 (130-300)	210 (150-370)	0.1
**CK-MB mass peak value (ng/ml)**	164 (60-290)	127 (47-252)	300 (165-493)	<0.001
**Anterior infarction, n (%)**	332 (50)	196 (39)	136 (84)	<0.001
**TIMI flow grade before PCI (%)**				0.2
**0**	345 (52)	252 (51)	93 (57)	
**1**	44 (7)	34 (7)	10 (6)	
**2**	73 (11)	52 (10)	21 (13)	
**3**	197 (30)	159 (32)	38 (24)	
**TIMI flow grade after PCI (%)**				0.04
**0**	17 (3)	15 (3)	2 (1)	
**1**	6 (1)	3 (1)	3 (2)	
**2**	50 (7)	30 (6)	20 (12)	
**3**	586 (89)	449 (90)	137 (85)	
**Grace Risk Score**	135±32	133±31	142±34	0.004
**Timi Risk Score**	2 (1-4)	2 (1-4)	3 (2-5.5)	<0.001

Abbreviations: CK-MB: creatine kinase myocardial band; PCI: primary coronary intervention; STEMI: ST-segment elevation myocardial infarction; TIMI: thrombolysis in myocardial infarction.

Table S3. Baseline characteristics of COVID-19 patients with bilateral pneumonia and without bilateral pneumonia.

	All	No bilateral pneumonia	Bilateral pneumonia	*p*-value
**Patients, n**	103	51	52	
**Age (years)**	69±16	73±16	65±14	0.006
**Male sex, n (%)**	49 (48)	19 (37)	30 (58)	0.04
**Diabetes mellitus, n (%)**	25 (24)	12 (24)	13 (25)	0.9
**Hypertension, n (%)**	57 (55)	30 (59)	27 (52)	0.5
**Hypercholesterolemia, n (%)**	42 (41)	22 (43)	20 (38)	0.4
**Smoker, n (%)**	5 (5)	4 (8)	1 (2)	0.1
**Heart rate (beats per minute)**	89±18	85±16	93±20	0.05
**Systolic blood pressure (mmHg)**	131±26	128±22	132±25	0.4
**Time to symptoms to first medical contact (min)**	5 (1-10)	4 (0-11)	7 (4-10)	0.05
**Creatinine (mg/dL)**	0.86 (0.74-1.11)	0.85 (0.70-1.12)	0.87 (0.79-1.07)	0.5
**Glucose (mg/dL)**	114 (97-135)	114 (97-136)	113 (98-133)	0.7
**Previous cardiovascular disease (%)**	24 (23)	14 (27)	10 (19)	0.4
**COPD (%)**	6 (6)	2 (4)	4 (8)	0.4
**D-dimer (ng/mL)**	602 (297-1248)	735 (330-1579)	544 (286-1089)	0.3
**GOT (U/L)**	35 (23-52)	31 (20-38)	40 (29-67)	0.001
**GPT (U/L)**	23 (14-44)	18 (11-27)	30 (20-60)	<0.001
**LDH (U/L)**	590±243	508±240	656±229	0.005
**CRP (mg/L)**	45 (10-88)	25 (5-61)	63 (32-119)	0.001
**Hydroxychloroquine (%)**	72 (70)	26 (51)	46 (88)	<0.001
**Azithromycin (%)**	63 (61)	26 (51)	37 (71)	0.03
**Tocilizumab (%)**	31 (30)	6 (12)	25 (48)	<0.001
**Corticoids (%)**	27 (26)	5 (10)	22 (42)	<0.001

Abbreviations: COPD: Chronic obstructive pulmonary disease; CRP: C-reactive protein; COVID-19: Coronavirus disease 2019; GOT: glutamic oxaloacetic transaminase; GPT: glutamate-pyruvate transaminase; LDH: Lactate dehydrogenase.

Table S4. Association between RALE score and the maximum neutrophil count, minimum lymphocyte count and maximum NLR in COVID-19 patients.

	**Mild (0 to 2)**	**Moderate (3 to 5)**	**Severe (6 to 8)**	**p-value**
**Maximum neutrophil count (x1000 cells(ml)**	7.6±4.1	8.4±4.4	14.0±6.3	<0.001
**Minimum lymphocyte count (x1000 cells(ml)**	1.3±0.6	0.9±0.6	0.6±0.3	<0.001
**Maximum NLR**	7.6±8.0	13.4±17.4	18.7±10.5	0.006

Abbreviations: COVID-19: Coronavirus disease 2019. NLR: neutrophil-to-lymphocyte ratio. RALE score: radiographic assessment of lung edema.

D. References

1. Bodí, V.; Sanchis, J.; López-Lereu, M.P.; Losada, A.; Núñez, J.; Pellicer, M.; Bertomeu, V.; Chorro, F.J.; Llácer, A. Usefulness of a comprehensive cardiovascular magnetic resonance imaging assessment for predicting recovery of left ventricular wall motion in the setting of myocardial stunning. *J. Am. Coll. Cardiol.* **2005**, *46*, 1747–1752, doi: 10.1016/j.jacc.2005.07.039.
2. Bodi, V.; Sanchis, J.; Nunez, J.; Mainar, L.; Lopez-Lereu, M.P.; Monmeneu, J. V; Rumiz, E.; Chaustre, F.; Trapero, I.; Husser, O.; et al. Prognostic value of a comprehensive cardiac magnetic resonance assessment soon after a first ST-segment elevation myocardial infarction. *JACC. Cardiovasc. Imaging* **2009**, *2*, 835–842, doi: 10.1016/j.jcmg.2009.03.011.
